# A comparative analysis of baseline and post-intervention surveillance in a hookworm control pilot: Evidence from Tiantai County, Zhejiang Province, 2022–2024

**DOI:** 10.1371/journal.pntd.0014835

**Published:** 2026-10-29

**Authors:** Qing Gao, Haiyan Hu, Junhua Ge, Weilong Pang, Yingjun Chen, Bingzhi Chen, Xiang Zheng, Weiwei Shen, Jimin Sun, Haijiang Lin

**Affiliations:** 1 Infectious Disease Prevention and Control Center, Taizhou City Center for Disease Control and Prevention, Taizhou, Zhejiang, China; 2 Department of Inspection and Testing, Tiantai County Center for Disease Control and Prevention, Taizhou, Zhejiang, China; 3 Tiantai County Center for Disease Control and Prevention, Taizhou, Zhejiang, China; 4 Department of Infectious Diseases, People’s Hospital of Tiantai County, Taizhou, Zhejiang, China; 5 Department of Microbiological Laboratory, Taizhou City Center for Disease Control and Prevention, Taizhou, Zhejiang, China; 6 Infectious Disease Prevention and Control Center, Zhejiang Center for Disease Control and Prevention, Hangzhou, Zhejiang, China; 7 Taizhou City Center for Disease Control and Prevention, Taizhou, Zhejiang, China; 8 Key Laboratory of Public Health Safety of the Ministry of Education, Fudan University, Shanghai, China; Washington University in St Louis School of Medicine, UNITED STATES OF AMERICA

## Abstract

Hookworm infection remains a neglected tropical disease in rural areas where low-level transmission may persist despite long-term control efforts. This repeated cross-sectional study evaluated changes in hookworm infection in six rural villages of Tiantai County, Zhejiang Province, China, before and after implementation of an integrated control program. In 2022, approximately 200 permanent residents aged ≥3 years were sampled from each village for the baseline survey. After one year of integrated interventions, including sanitation improvement, health education, deworming, and strengthened village-level management, another cross-sectional survey was conducted in the same villages in 2024. Hookworm infection was detected using stool examination. Prevalence was compared between survey years, and Firth penalized logistic regression was used to estimate adjusted associations with infection while addressing sparse data and complete separation. A total of 1,218 individuals were examined in 2022, of whom 58 were positive for hookworm infection, corresponding to a prevalence of 4.76%. In 2024, 1,222 individuals were examined and 13 infections were detected, corresponding to a prevalence of 1.06%. The overall prevalence decreased by 77.73%. In the Firth penalized logistic regression model adjusted for sex, age group, educational level, occupation, and village, the odds of infection in the 2024 post-intervention survey were significantly lower than those in the 2022 baseline survey (adjusted *OR* = 0.223, 95% *CI*: 0.125–0.399, *P* < 0.001). Male sex, primary education or below, and residence in Qianyang, Xiatang, or Xiaqiao villages were associated with higher infection odds. Hookworm prevalence declined substantially in the same villages after implementation of integrated control measures. However, the absence of non-intervention control villages limits causal attribution. Sustained surveillance and targeted interventions remain needed for high-risk populations and villages.

## Author summary

Hookworm infection is a parasitic disease that can cause anemia, fatigue, and poor nutrition, especially in rural communities where people often work in close contact with soil. Although national control programs have reduced infection in many places, some villages and population groups can remain at risk, and it is not always clear which measures work best in real-world settings. In this study, we compared community surveys conducted in Tiantai County, Zhejiang Province, in 2022 and again in 2024 after implementation of an integrated hookworm control pilot that included sanitation improvement, health education, and deworming treatment. The overall proportion of people with hookworm infection fell from 4.76% to 1.06%, and most baseline-positive individuals who were successfully followed became infection-free. However, infection did not disappear completely. However, this was not a randomized or controlled study, and not all baseline-positive individuals were followed; therefore, the reduction cannot be attributed solely to the intervention package. Older adults, people with lower levels of schooling, and residents in certain villages continued to have higher risk. These findings suggest that integrated approaches may help reduce hookworm transmission, but long-term success requires better monitoring of intervention coverage, behavioral practices, environmental conditions, and residual high-risk groups.

## Introduction

Hookworm disease is a soil-transmitted parasitic infection caused by hematophagous nematodes that reside in the human small intestine. Infection occurs primarily through percutaneous or oral exposure to contaminated soil, and humans are generally susceptible to the disease [1]. Once infected, adult hookworms attach to the intestinal mucosa and feed on blood for prolonged periods, leading to gastrointestinal dysfunction, iron-deficiency anemia, protein-energy malnutrition, and reduced physical capacity [2]. In 2010, the World Health Organization (WHO) classified hookworm infection as one of the most important neglected tropical diseases (NTDs), with more than 500 million people affected worldwide [3]. In China, hookworm disease is among the five major parasitic diseases and contributes substantially to the national burden of anemia [4]. Each adult hookworm may cause approximately 0.2 mL of blood loss per day, and an infection burden of 40–160 worms can result in typical microcytic hypochromic iron-deficiency anemia [5]. Globally, hookworm infection is estimated to account for approximately 845,000 disability-adjusted life years (DALYs) annually [6], imposing a considerable burden on public health and socioeconomic development.

Hookworm disease is widely distributed across tropical and subtropical regions, with the highest prevalence observed in impoverished areas of developing countries such as South Asia, Southeast Asia, sub-Saharan Africa, and Latin America. Poor sanitation, land-use patterns, and behavioral factors collectively facilitate ongoing transmission in these settings [7]. In China, hookworm infection is reported in most provinces except for some arid regions in the northwest. According to the 2021 national surveillance of soil-transmitted helminthiases, Zhejiang Province reported the fourth-highest hookworm infection rate nationwide (2.20%), significantly above the national average of 0.67%, suggesting that the province remains at relatively high risk for hookworm transmission.

The transmission and distribution of hookworm infection are influenced by multiple factors, including environmental conditions, climate, occupational exposure, hygiene behaviors, educational attainment, and regional socioeconomic status [8]. Rural populations—particularly farmers—constitute the primary high-risk group, as they frequently engage in agricultural activities, work barefoot, and have repeated contact with contaminated soil [9]. Although China has achieved substantial reductions in hookworm prevalence through rural sanitation improvements, health education campaigns, and large-scale deworming interventions, some regions continue to exhibit “residual hotspots,” higher infection rates among older adults, and localized clusters of transmission [10]. In this context, repeated surveys in the same region can describe temporal changes after integrated control measures and identify residual high-risk populations. However, in the absence of an untreated comparison group, such surveys should be interpreted as programmatic surveillance rather than as definitive causal evaluations.

## Results

### Total infection rate

To describe post-intervention changes in hookworm infection, cross-sectional surveys were conducted in six selected villages in 2022 (baseline) and 2024 (post-intervention). In 2022, a total of 1,218 individuals were enrolled, including 567 males and 651 females, with 58 hookworm infections detected, yielding an infection rate of 4.76% (58/1218). After implementation of integrated control measures in 2023, 1,222 individuals were included in the 2024 follow-up survey (573 males, 649 females), and 13 hookworm infections were identified, resulting in an infection rate of 1.06% (13/1222). Compared with baseline, the overall infection rate decreased by 77.73%. Except for Tunqiao Village, where no infections were detected in either survey, all other villages achieved the target reduction of at least 50% relative to baseline. The decline in infection rate between the two surveys was statistically significant (χ^2^ = 28.23, *P* < 0.001). Because the study lacked a non-intervention control group, this reduction should be interpreted as a post-intervention decline rather than as definitive evidence that the intervention package alone caused the decline.

Considering potential sampling variation and population mobility between the two surveys, a longitudinal descriptive analysis was conducted among individuals who participated in both rounds (n = 266). Among the 58 baseline-positive cases, 36 (62.1%) were successfully followed up. Of these followed individuals, 34 became egg-negative, corresponding to an egg-negative conversion proportion of 94.4% (34/36). The remaining two cases did not completely clear infection but showed marked reductions in EPG counts: one decreased from 816 to 72 EPG (91.2% reduction), and the other from 264 to 192 EPG (27.3% reduction). Because 22 baseline-positive individuals were not followed, this estimate may be affected by selection bias and should not be interpreted as a population-level cure rate (Table 1).

**Table 1:** Hookworm infection rate among rural population in Tiantai County in 2022 and 2024.

Region	2022			2024			Dual-positive Cases	Overlapping Sample	Infection Decline Rate (%)
	Number of investigations	Number of infections	Infection rate (%)	Number of investigations	Number of infections	Infection rate (%)			
Chishan Village	211	5	2.37	208	0	0.00	0	49	100
Tunqiao Village	202	0	0	202	0	0.00	0	6	/
Xiatang Village	200	15	7.50	203	2	0.99	1	118	86.80
Qianyang Village	201	22	10.95	201	6	2.99	1	74	72.69
Xiaqiao Village	203	13	6.40	206	4	1.94	0	12	69.69
Shanglu Village	201	3	1.49	202	1	0.50	0	7	66.44
Total	1218	58	4.76	1222	13	1.06	2	266	77.73

### Regional distribution

Among the 1,218 stool samples collected in 2022, 58 were positive for hookworm eggs, with a larval culture positivity rate of 80.49%; all larvae were identified as *Necator americanus*. Among the six villages, Qianyang Village had the highest infection rate (10.95%, 22/201), followed by Xiatang Village (7.50%, 15/200), whereas Tunqiao Village had no detected infections (0/202). Differences in infection rates among villages were statistically significant (χ^2^ = 38.959, *P* < 0.001).

In 2024, 13 hookworm infections were detected among 1,222 samples, with a larval culture positivity rate of 100%, and all were *N. americanus*. Infection rates decreased markedly across all villages and fell below 3%. Notably, infection rates decreased from 10.95% to 2.99% in Qianyang Village, from 7.50% to 0.99% in Xiatang Village, and from 6.40% to 1.94% in Xiajiao Village. Although inter-village differences in infection rates remained statistically significant in 2024 (Fisher’s exact test = 11.360, *P* = 0.01), the magnitude of differences was substantially reduced compared with 2022 (Fig 1).

**Fig 1:**
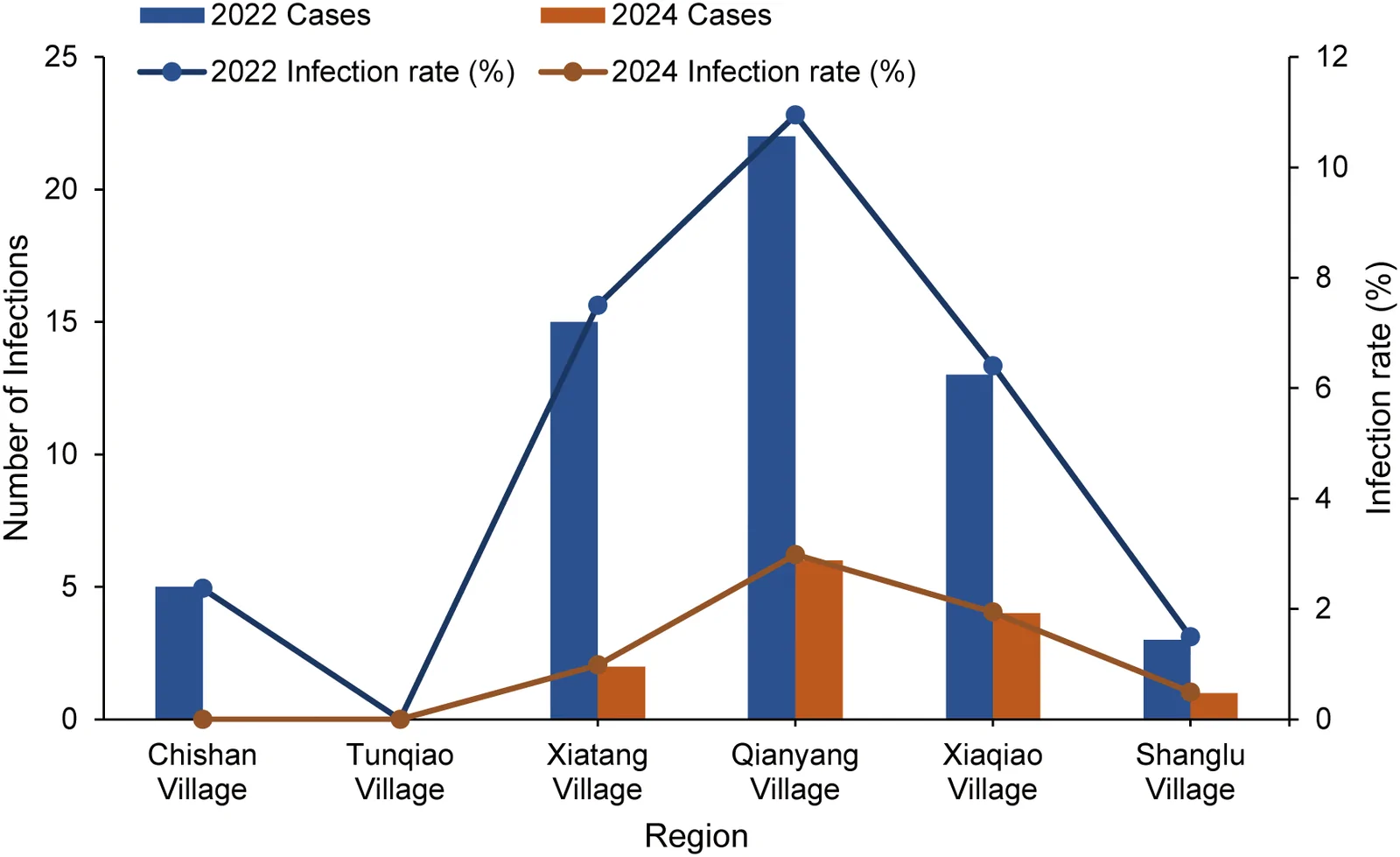
Infection status of hookworms in different regions of Tiantai County in 2022 and 2024.

### Gender distribution

In 2022, 31 hookworm infections were identified in males (5.47%, 31/567) and 27 in females (4.15%, 27/651), with no statistically significant difference between sexes (χ^2^ = 1.164, *P* = 0.281). In 2024, the infection rate among males was 1.75% (10/573), compared to 0.46% (3/649) among females. The higher infection rate in males was statistically significant (χ^2^ = 4.759, *P* = 0.029). Between 2022 and 2024, the infection rate in males decreased from 5.47% to 1.75% (68.0% reduction), and in females from 4.15% to 0.46% (88.9% reduction). The declines in both sexes were statistically significant (χ^2^ = 11.388, *P* = 0.001; χ^2^ = 19.578, *P* < 0.001).

### Age distribution

In 2022, no infections were detected among participants aged 3–40 years, whereas the infection rate increased steadily with age among individuals aged ≥41 years (χ^2^_*trend*_ = 4.336, *P* < 0.05). Participants older than 80 years had the highest infection rate (10.94%). In 2024, infection rates decreased across all age groups: infection in individuals >80 years declined from 10.94% to 1.25%; in the 71–80 years group from 6.51% to 1.00%; and in the 51–60 and 61–70 years groups by 61.9% and 79.2%, respectively. Because no infection was observed among individuals aged 3–40 years, age-specific risk estimates for this group could not be calculated, and education-specific infection risk among participants under 40 years could not be meaningfully modeled (Fig 2).

**Fig 2:**
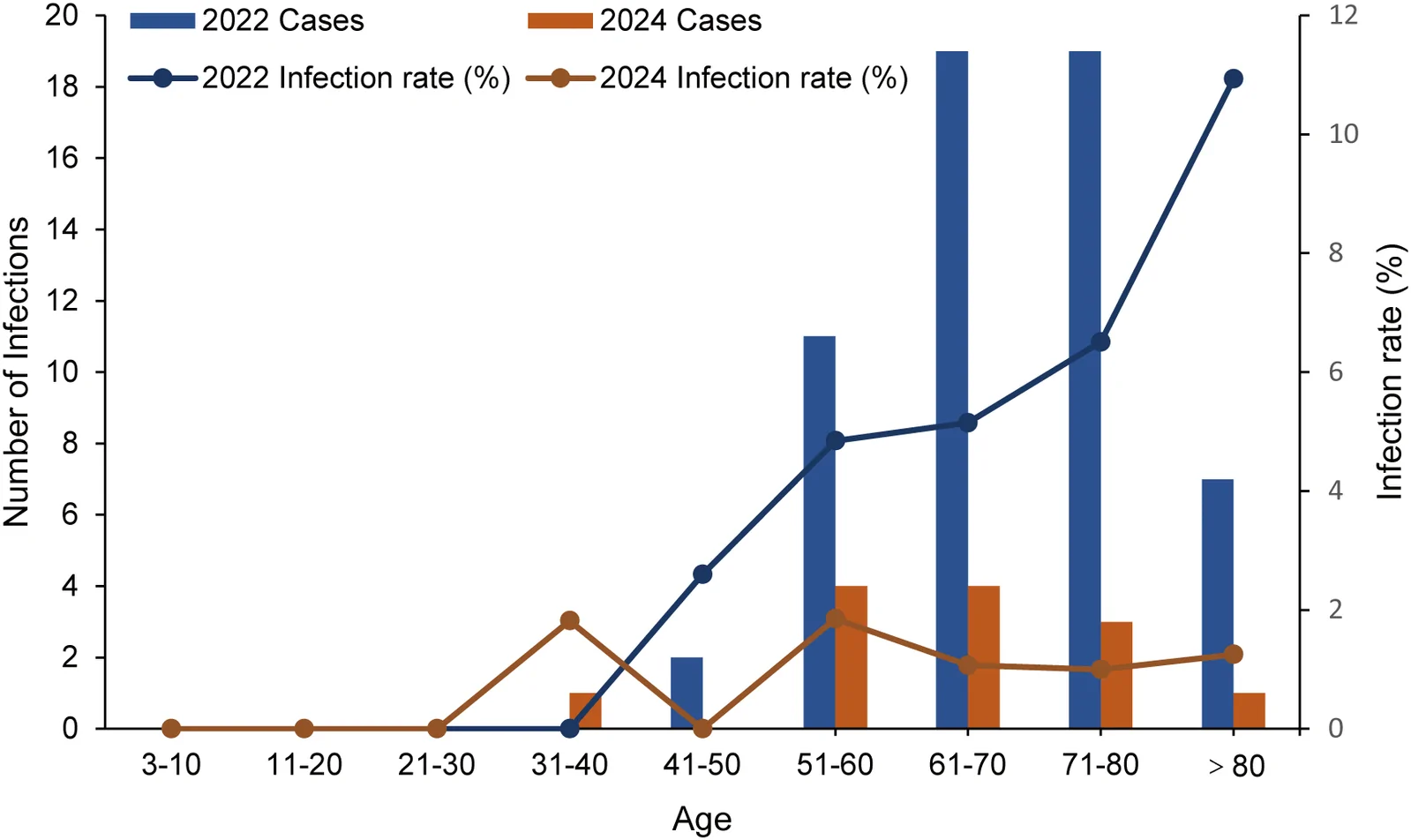
Infection status of hookworms in different age groups in Tiantai County in 2022 and 2024.

### Occupational and educational level distribution

In both survey years, all detected hookworm infections occurred among farmers. The infection rate among farmers decreased from 5.29% (58/1097) in 2022 to 1.16% (13/1117) in 2024, representing a 78.07% reduction (χ^2^ = 30.314, *P* < 0.001).

In 2022, infection rates were inversely associated with educational level: 6.22% among participants with primary education or below, 3.04% among those with middle school, 1.54% with high school, and 0% among those with college or above. Differences across educational groups were statistically significant (Fisher’s exact test = 7.275, *P* = 0.005). Infection rates decreased across all educational groups in 2024: to 1.72% in the primary-or-below group, 0.44% in the middle school group, and 0% in the high school or above group. Longitudinal comparisons showed reductions of 72.4% and 85.5% in the primary-or-below and middle school groups, respectively. For participants younger than 40 years, no hookworm infections were detected in either survey; therefore, educational level in this age stratum could be summarized only descriptively and was not included as a separate stratified risk model (S1 Table).

### Firth penalized logistic regression analysis

To address sparse data and complete or quasi-complete separation, Firth penalized logistic regression was performed. In the full model including occupation, the odds of infection in the 2024 survey were significantly lower than in the 2022 survey after adjustment for sex, age group, educational level, occupation, and village (adjusted *OR* = 0.223, 95% *CI*: 0.125–0.399, *P* < 0.001). Male participants had higher odds of infection than females (adjusted *OR* = 1.792, 95% *CI*: 1.104–2.909, *P* = 0.018). Participants with primary education or below had higher odds than those with middle school education (adjusted *OR* = 2.235, 95% *CI*: 1.206–4.139, *P* = 0.011). Farmer occupation showed an elevated odds ratio compared with non-farm1er occupation, but the confidence interval was wide and the association did not reach conventional statistical significance (adjusted *OR* = 12.630, 95% *CI*: 0.820–194.578, *P* = 0.069).

Village-level differences remained after adjustment. Compared with Shanglu Village, the odds of infection were significantly higher in Qianyang Village (adjusted *OR* = 7.588, 95% *CI*: 2.809–20.501, *P* < 0.001), Xiatang Village (adjusted *OR* = 3.287, 95% *CI*: 1.170–9.231, *P* = 0.024), and Xiaqiao Village (adjusted *OR* = 4.431, 95% *CI*: 1.582–12.413, *P* = 0.005). Chishan Village and Tunqiao Village did not differ significantly from Shanglu Village in the adjusted model (Table 2).

**Table 2:** Multivariable Firth penalized logistic regression analysis of hookworm infection, including occupation.

Variable	Reference	*Adjusted OR*	95%*CI*	P value
Year				
2024	2022	0.223	0.125–0.399	<0.001
Sex				
Male	Female	1.792	1.104–2.909	0.018
Occupation				
Farmer	Non-farmer	12.630	0.820–194.578	0.069
Educational level				
Primary school or below	Middle school	2.235	1.206–4.139	0.011
High school		0.746	0.193–2.890	0.672
College or above		1.331	0.077–22.936	0.844
Age				
51-60	≤50	2.420	0.781–7.500	0.126
61-70		1.841	0.611–5.547	0.278
71-80		2.124	0.692–6.522	0.188
>80		3.115	0.874–11.096	0.080
Region				
Qianyang Village	Shanglu Village	7.588	2.809–20.501	<0.001
Xiatang Village		3.287	1.170–9.231	0.024
Chishan Village		1.681	0.488–5.782	0.410
Tunqiao Village		0.119	0.007–2.039	0.142
Xiaqiao Village		4.431	1.582–12.413	0.005

Note. *OR* = odds ratio; *CI* = confidence interval. The model included survey year, sex, occupation, educational level, age group, and village. Firth penalized logistic regression was used because sparse positive events and complete or quasi-complete separation were present in several categories. The occupation estimate should be interpreted cautiously because no infections occurred among non-farmers.

Because all infected individuals were farmers, a sensitivity analysis excluding occupation was also conducted. The association between survey year and infection remained similar (adjusted *OR* for 2024 vs 2022 = 0.213, 95% *CI:* 0.119–0.382, *P* < 0.001). In this sensitivity model, older age groups were significantly associated with higher infection odds, and the associations for sex, primary-or-below education, Qianyang Village, Xiatang Village, and Xiaqiao Village remained consistent with the full model (S2 Table). These findings indicate that the observed reduction in infection was robust to model specification, while the effect of age should be interpreted in relation to occupational exposure and sparse outcome events.

## Discussion

Based on baseline data collected in 2022 and follow-up findings from 2024, this study systematically evaluated the epidemiological characteristics of hookworm infection and post-intervention changes in Tiantai County, Zhejiang Province. The two cross-sectional surveys demonstrated that the overall infection rate declined to 1.06% in 2024, representing a 77.73% reduction compared with baseline. Among the followed subset of baseline-positive individuals, 94.4% became egg-negative after intervention. However, because the study did not include a non-intervention control group and because only 36 of 58 baseline-positive individuals were followed, these findings should be interpreted as evidence of a substantial post-intervention decline rather than as definitive proof of intervention effectiveness.

The reduction in infection rate and narrowing of inter-village disparities in 2024 are consistent with the national efforts to promote rural sanitation improvements, including the “toilet revolution,” enhanced sewage management, and strengthened health education initiatives [11]. At baseline, Necator americanus was the predominant species. Although local soil moisture, drainage, and behavioral exposure were not directly measured in this study, N. americanus larvae are known to survive more readily in warm and humid environments, and sanitation and behavior change can reduce soil contamination and percutaneous exposure pathways [12]. Similar outcomes have been reported elsewhere, where improvements in sanitation infrastructure—such as access to tap water and increased coverage of sanitary latrines—were associated with lower hookworm infection risk [13,14]. Chemotherapy may also have contributed to infection reduction. According to WHO guidelines, a single dose of albendazole can reduce hookworm prevalence substantially [15]; the 94.4% cure rate among infected individuals in our core cohort aligns with documented treatment efficacy. The high egg-negative conversion proportion among the followed infected individuals in this study is compatible with treatment effects, but the incomplete follow-up means that it should be interpreted cautiously.

Although infection rates fell below 3% across all six villages in 2024, regional disparities persisted. The infection risk of Qianyang Village, Xiatang Village, and Xiaqiao Village is still significantly higher than that of Shanglu Village in the multi factor model, and the characteristic of “hotspot concentration” has also been reported by other scholars [16]. Possible explanations include: (1) higher soil moisture and poor drainage, which favor larval survival; (2) agricultural practices involving waterlogged or paddy fields, where barefoot labor is more common; (3) suboptimal use or maintenance of sanitary latrines; and (4) inadequate penetration of health education. Evidence suggests that differences in sanitation conditions and behavioral practices are major determinants of post-intervention infection variability Because this study did not directly measure village-level environmental contamination, drainage conditions, footwear use, latrine-use compliance, or health education coverage, the reasons for persistent village-level differences cannot be determined from the present data. Plausible explanations include differences in agricultural exposure, environmental suitability for larval survival, sanitation infrastructure, and uptake of health education, but these should be regarded as hypotheses requiring future verification [17]. These findings highlight the need for sustained, village-level precision management and improved monitoring of intervention coverage in high-risk communities.

In 2022, hookworm infection exhibited a typical age-related pattern, increasing markedly among individuals aged ≥41 years, a trend consistent with national surveillance data [18]. By 2024, infection rates declined substantially across age groups, with a notable reduction in individuals aged ≥80 years. Nonetheless, age remained an independent risk factor with infection. Possible explanations include: (1) greater frequency of soil-related labor exposure among older adults, including barefoot agricultural work—identified as a major risk factor for hookworm infection [19]; (2) age-related decline in immune function, making persistent infection more likely; and (3) limited receptiveness to health education, resulting in less behavioral change. Therefore, elderly populations should remain a priority for future interventions.

Although no significant sex difference was observed in 2022, by 2024 the infection rate among females (0.46%) was lower than that among males (1.75%). This pattern may reflect gender-related differences in agricultural labor and soil exposure, but the present study did not collect detailed occupational task or behavior data to confirm this mechanism. A national analysis of soil-transmitted helminths (STH) from 1990 to 2021 similarly reported persistently higher age-standardized prevalence in males than females for hookworm and other STH infections [20]. This may reflect greater agricultural exposure among men, as well as higher diagnosis rates associated with better health care access in certain settings [21]. These findings suggest that male farmers may require continued attention in health education and follow-up screening, while future evaluations should include direct measures of exposure behavior.

Participants with primary education or below had the highest infection risk, and multivariate analysis showed their risk to be nearly twice that of those with middle school. Similar findings have been reported in Shanghai, Shandong, and other regions, where lower educational attainment was associated with higher hookworm prevalence, potentially through differences in health literacy, hygiene practices, and occupational exposure [22,23]. Although infection rates declined in all education groups after intervention, the primary-education-or-below group remained at relatively higher risk, suggesting that educational level represents an important social determinant of residual hookworm infection. For individuals younger than 40 years, no infections were detected, so the present data cannot evaluate education-specific infection risk within that age stratum. Sustained health education targeting low-literacy populations is therefore essential.

The use of Firth penalized logistic regression strengthened the statistical analysis by addressing the sparse data structure and complete separation noted in conventional logistic regression. In this dataset, all infected individuals were farmers, and some categories such as Tunqiao Village and college-or-above education had zero or very few positive cases. Under such conditions, maximum-likelihood logistic regression may yield unstable coefficients, inflated odds ratios, or infinite confidence intervals. The Firth model provided finite estimates and allowed occupation and low-frequency categories to be retained in the multivariable analysis. The sensitivity model excluding occupation produced a similar estimate for survey year, indicating that the lower infection odds in 2024 was not dependent on whether occupation was included in the model.

The results suggest that residual hookworm infection was concentrated in specific population groups. Men had higher adjusted odds of infection than women, which may reflect differences in agricultural labor patterns, soil contact, and protective behaviors. Participants with primary education or below also had higher infection odds, suggesting that educational attainment remains an important social determinant of infection risk. Lower education may be associated with limited awareness of transmission routes, weaker adoption of protective practices, or greater involvement in high-exposure agricultural activities [24]. These findings support the need for health education that is adapted to low-literacy populations rather than relying only on general publicity campaigns.

Age-related patterns require careful interpretation. Descriptively, prevalence was higher among older adults, and the sensitivity model excluding occupation showed significantly increased odds across older age groups. However, after occupation was included in the full Firth model, age-group estimates remained above 1 but were no longer statistically significant. This attenuation suggests that the apparent age effect may be partly explained by occupational exposure, because older residents in these villages may be more likely to remain engaged in farming or soil-contact activities. Therefore, older adults should still be considered a priority group for targeted surveillance and health education, but the mechanism should be framed as a combination of age, occupation, and exposure opportunity rather than age alone.

Village-level heterogeneity persisted despite the overall decline in prevalence. Qianyang, Xiatang, and Xiaqiao villages had significantly higher adjusted odds of infection than Shanglu Village, indicating that local transmission risk was not completely eliminated. Because this study did not directly measure behavioral or environmental variables, the underlying causes of these village differences cannot be determined from the present data. Plausible explanations include differences in agricultural production patterns, frequency of soil contact, sanitation facility use, environmental humidity and drainage conditions, and intensity of health education implementation. Future evaluations should include direct measures of barefoot farming, latrine use, soil exposure, anthelmintic compliance, and environmental contamination to clarify the pathways driving residual village-level risk.

Several limitations should be acknowledged. First, the study used a before-and-after repeated cross-sectional design without a non-intervention comparison group; therefore, secular trends, population mobility, concurrent sanitation improvements, or other public health activities may have contributed to the observed decline. Second, most participants differed between the two surveys, and the overlapping individuals should be considered only a supplementary subset rather than a formal longitudinal cohort. Third, behavioral and environmental risk factors were not collected at the individual level, limiting causal interpretation of the observed associations. Fourth, despite the use of Firth penalized regression, the number of positive cases in 2024 was small, and estimates for categories with zero or very few infections should be interpreted cautiously. These limitations were addressed by using conservative causal language, reporting sensitivity analyses, and emphasizing the need for future studies with control villages and more detailed exposure measurements.

## Conclusions

In this repeated cross-sectional surveillance study, hookworm infection in Tiantai County declined substantially after implementation of integrated control measures. The decline is consistent with the expected benefits of sanitation improvement, health education, and deworming, but the absence of a control group means that causality cannot be established definitively. Elderly individuals, populations with lower educational attainment, male participants, and residents of specific high-risk villages remained more vulnerable to infection. Future control efforts should prioritize precision management in residual high-risk communities, expand health education targeting older adults and low-literacy groups, strengthen sanitation infrastructure, and incorporate direct monitoring of behavior, environmental contamination, and intervention coverage to support sustainable reduction of hookworm disease burden.

## Methods

### Ethics approval

This study was reviewed and approved by the ethics Review committee of Taizhou City Center for Disease Control and Prevention [TZCDC-2026-1IR-001]. Written informed consent was obtained from all adult participants. For participants aged <18 years, written informed consent was obtained from a parent or legal guardian, and assent was obtained from the child or adolescent when appropriate. All data were anonymized before analysis.

### Setting

Tiantai County, located in Taizhou City of Zhejiang Province, lies in the east-central region of the province. The terrain is predominantly composed of low mountains and hills, accounting for approximately 81% of the county’s total area. The region has a mid-subtropical monsoon climate characterized by four distinct seasons, abundant precipitation, and adequate heat.

### Data sources

In accordance with the *Hookworm Control Pilot Program of Zhejiang Province*, six villages with typical agricultural production characteristics were selected in Tiantai County: Chishan and Tunqiao villages in Pingqiao Town, Xiatang and Qianyang villages in Nanping Township, Xiajiao Village in Sanhe Town, and Shanglu Village in Baihe Town. The target sample size of at least 200 permanent residents per village was based on the provincial pilot surveillance protocol and operational feasibility for village-level monitoring, rather than on a formal randomized trial power calculation. Eligible participants were permanent residents aged ≥3 years who had lived in the village for at least six months. Cluster sampling was used to include children, adolescents, adults, and older adults.

A standardized questionnaire designed by the Zhejiang Provincial Center for Disease Control and Prevention was used to collect demographic information, including sex, age, occupation, educational level, and history of anthelmintic medication use. Direct behavioral and environmental measurements, such as footwear use, frequency of barefoot agricultural work, latrine-use compliance, soil contamination, and village-level sanitation coverage, were not included in the analytical dataset. A baseline survey was conducted in 2022, followed by a post-intervention survey in 2024 after one year of integrated control measures.

### Main interventions

After the baseline survey, a one-year comprehensive prevention and control strategy was implemented in the target area in 2023. This strategy focuses on “health education as the guide and infectious source control as the main focus”, integrating four major modules: environmental modification, behavioral intervention, drug treatment, and key management. First, the program promoted the construction and renovation of harmless sanitary toilets and improvement of qualified rural drinking-water coverage to reduce environmental contamination. Second, multi-level health education was conducted through lectures, household visits, and distribution of educational materials to improve knowledge of parasite life cycles, personal protection, and sanitary toilet use. Third, standardized deworming drugs, such as albendazole, were provided to baseline infected individuals and selected high-risk populations according to the local control protocol. Fourth, villages with higher baseline infection rates and less favorable environmental conditions were selected for strengthened management, including more frequent health education and environmental clean-up. Because all six villages received the integrated package and no untreated control village was available, intervention exposure was collinear with survey year and could not be entered as a separate covariate in the multivariable model. In addition, standardized quantitative coverage indicators for each intervention component were not available; therefore, intervention implementation was described qualitatively and the results were interpreted as post-intervention changes rather than proof of causality for any single component.

### Research methods

Fresh stool samples (>30 g) were collected from all participants. Hookworm eggs were detected using the modified Kato–Katz thick-smear technique [25], with two slides prepared per sample. Other helminth eggs identified during testing were also recorded. Infection intensity was classified based on the number of eggs per gram (EPG) of feces: < 2000 EPG as light infection, 2000–3999 EPG as moderate infection, and ≥4000 EPG as heavy infection. Individuals who tested positive for hookworm eggs underwent additional stool culture using the Harada–Mori filter paper strip method to obtain filariform larvae, followed by species identification.

All data were organized using Excel, and statistical analyses were performed with R (version 4.3.2). Descriptive analyses of demographic characteristics (sex, age group, educational level, and village) were conducted. Categorical variables were expressed as frequencies and proportions. The chi-square test was used to compare proportions between groups; for ordinal variables such as age, chi-square or trend chi-square tests were applied. Infection rate was calculated as “number of hookworm egg–positive individuals/ total number of individuals examined × 100%.”

To identify factors associated with hookworm infection while addressing sparse data and complete or quasi-complete separation, Firth penalized logistic regression was used as the primary multivariable analysis. Hookworm infection status was used as the dependent variable, and survey year, sex, age group, educational level, occupation, and village were included as independent variables. The 2022 survey, female sex, age ≤ 50 years, middle school education, non-farmer occupation, and Shanglu Village were used as reference categories. Adjusted odds ratios and 95% confidence intervals were estimated using profile penalized likelihood.

Firth penalized regression was selected because several categories had zero or very few positive cases. In particular, all hookworm-positive individuals were farmers, whereas no infections were detected among non-farmers, resulting in complete separation in conventional maximum-likelihood logistic regression. Some educational and village categories also had sparse positive events. Under these conditions, conventional logistic regression may produce unstable coefficients, inflated odds ratios, or infinite confidence intervals, whereas Firth penalized regression provides finite and less biased estimates.

Because occupation showed complete separation and may be closely related to age and village-level exposure patterns, a sensitivity analysis excluding occupation was also conducted. The consistency of the survey-year effect and other covariate estimates between the full model and the sensitivity model was used to evaluate the robustness of the findings.

All statistical tests were two-sided, and *P* < 0.05 was considered statistically significant.

## Supporting information

S1 Table Educational distribution and hookworm infection status among participants aged <40 years in 2022 and 2024. (DOCX)

S2 Table Sensitivity analysis using Firth penalized logistic regression excluding occupation. (DOCX)

S1 Data Case Information Form. (XLSX)
