# Supplementary material for: A comparative analysis of baseline and post-intervention surveillance in a hookworm control pilot: Evidence from Tiantai County, Zhejiang Province, 2022–2024
Source: PLoS Negl Trop Dis. 2026 Oct 29;20(10):e0014835. doi: 10.1371/journal.pntd.0014835 (PMC13718970; doi:10.1371/journal.pntd.0014835)
Supplement: Supplementary file 1 [file pntd.0014835.s001.docx]

S1 Table . Educational distribution and hookworm infection status among participants aged <40 years in 2022 and 2024.

| Survey year | Participants aged <40 years | Primary or below | Middle school | High school | College or above | Hookworm-positive cases |
| --- | --- | --- | --- | --- | --- | --- |
| 2022 | 183 | 97 (53.0%) | 53 (29.0%) | 24 (13.1%) | 9 (4.9%) | 0 |
| 2024 | 177 | 54 (30.5%) | 47 (26.6%) | 49 (27.7%) | 27 (15.3%) | 0 |
| Total | 360 | 151 (41.9%) | 100 (27.8%) | 73 (20.3%) | 36 (10.0%) | 0 |
