# Supplementary material for: A comparative analysis of baseline and post-intervention surveillance in a hookworm control pilot: Evidence from Tiantai County, Zhejiang Province, 2022–2024
Source: PLoS Negl Trop Dis. 2026 Oct 29;20(10):e0014835. doi: 10.1371/journal.pntd.0014835 (PMC13718970; doi:10.1371/journal.pntd.0014835)
Supplement: Supplementary file 2 [file pntd.0014835.s002.docx]

S2 Table . Sensitivity analysis using Firth penalized logistic regression excluding occupation.

| Variable | Reference | *Adjusted OR* | 95%*CI* | *P* value |
| --- | --- | --- | --- | --- |
| Year |  |  |  |  |
| 2024 | 2022 | 0.213 | 0.119–0.382 | <0.001 |
| Sex |  |  |  |  |
| Male | Female | 1.722 | 1.061–2.795 | 0.028 |
| Educational level |  |  |  |  |
| Primary school or below | Middle school | 2.050 | 1.113–3.775 | 0.021 |
| High school |  | 0.812 | 0.208–3.173 | 0.764 |
| College or above |  | 1.037 | 0.053–20.248 | 0.981 |
| Age |  |  |  |  |
| 51-60 | ≤50 | 5.505 | 1.765–17.170 | 0.003 |
| 61-70 |  | 4.283 | 1.424–12.881 | 0.010 |
| 71-80 |  | 4.909 | 1.613–14.941 | 0.005 |
| ＞80 |  | 7.453 | 2.121–26.186 | 0.002 |
| Region |  |  |  |  |
| Qianyang Village | Shanglu Village | 7.257 | 2.683–19.629 | <0.001 |
| Xiatang Village |  | 3.421 | 1.216–9.624 | 0.020 |
| Chishan Village |  | 1.617 | 0.468–5.582 | 0.447 |
| Tunqiao Village |  | 0.120 | 0.007–2.065 | 0.144 |
| Xiaqiao Village |  | 4.307 | 1.535–12.086 | 0.006 |

Note. *OR* = odds ratio; *CI* = confidence interval. This sensitivity model excluded occupation because all infected individuals were farmers. The association between survey year and infection remained similar to the full model.
